# Small bowel obstruction caused by a true ileo-ileal knot: a rare case successfully treated by prior ligation of mesenteric vessels

**DOI:** 10.1186/s40792-021-01276-7

**Published:** 2021-08-26

**Authors:** Kohei Kanamori, Kazuo Koyanagi, Hitoshi Hara, Kenji Nakamura, Kazuhito Nabeshima, Miho Yamamoto, Yamato Ninomiya, Tadashi Higuchi, Kentaro Yatabe, Mika Ogimi, Kohei Tajima, Masaki Mori, Seiichiro Yamamoto, Toshio Nakagohri, Soji Ozawa

**Affiliations:** Department of Gastroenterological Surgery, Tokai University School of Medicine, 143 Shimokasuya, Isehara, Kanagawa 259-1193 Japan

**Keywords:** Ileo-ileal knot, Bowel knot, True knot, Small bowel obstruction

## Abstract

**Background:**

Intestinal knot formation, in which two segments of the intestine become knotted together, can result in intestinal obstruction. An ileo-ileal knot refers to knot formation between two ileal segments and is a very rare benign disease. We report a case of strangulated bowel obstruction caused by true ileo-ileal knot formation.

**Case presentation:**

An 89-year-old woman was referred to our hospital with the diagnosis of intestinal obstruction. Contrast-enhanced computed tomography revealed the small bowel forming a closed loop, with poor contrast effect. Based on the findings, the patient was diagnosed as having strangulated bowel obstruction, and emergency surgery was performed. At laparotomy, two segments of the ileum were found to be tied together forming a knot, and both segments were necrotic. Although it was necessary to release the strangulated small bowel, we did not immediately release the knot, but first proceeded with ligation of the mesenteric vessels to the strangulated small bowel to prevent dissemination of toxic substances from the necrotic bowel into the systemic circulation. The surgery was completed with resection of the necrotic ileum and anastomosis of the small intestine. The postoperative course was uneventful, and the patient was discharged home.

**Conclusion:**

We encountered a case of strangulated bowel obstruction caused by true ileo-ileal knot formation. Resection of the necrotic small intestine without releasing the knot could be performed safely, and might be considered as an option of surgical procedure.

## Background

Intestinal knot formation is the condition, where two intestinal segments become tied together to form a knot, resulting in intestinal obstruction with impaired blood flow. Almost all intestinal knot formation are found between the sigmoid colon and ileum, whereas knot formation between two segments of the ileum, the so-called “ileo-ileal knot,” is rare [1]. There have been only a few reports of ileo-ileal knots, and their etiology and risks remain unclear. Herein, we report a case of strangulated bowel obstruction caused by true ileo-ileal knot formation. We then summarize the reports on ileo-ileal knots and discuss the causes, treatment details, and treatment results.

## Case presentation

**Fig. 1:**
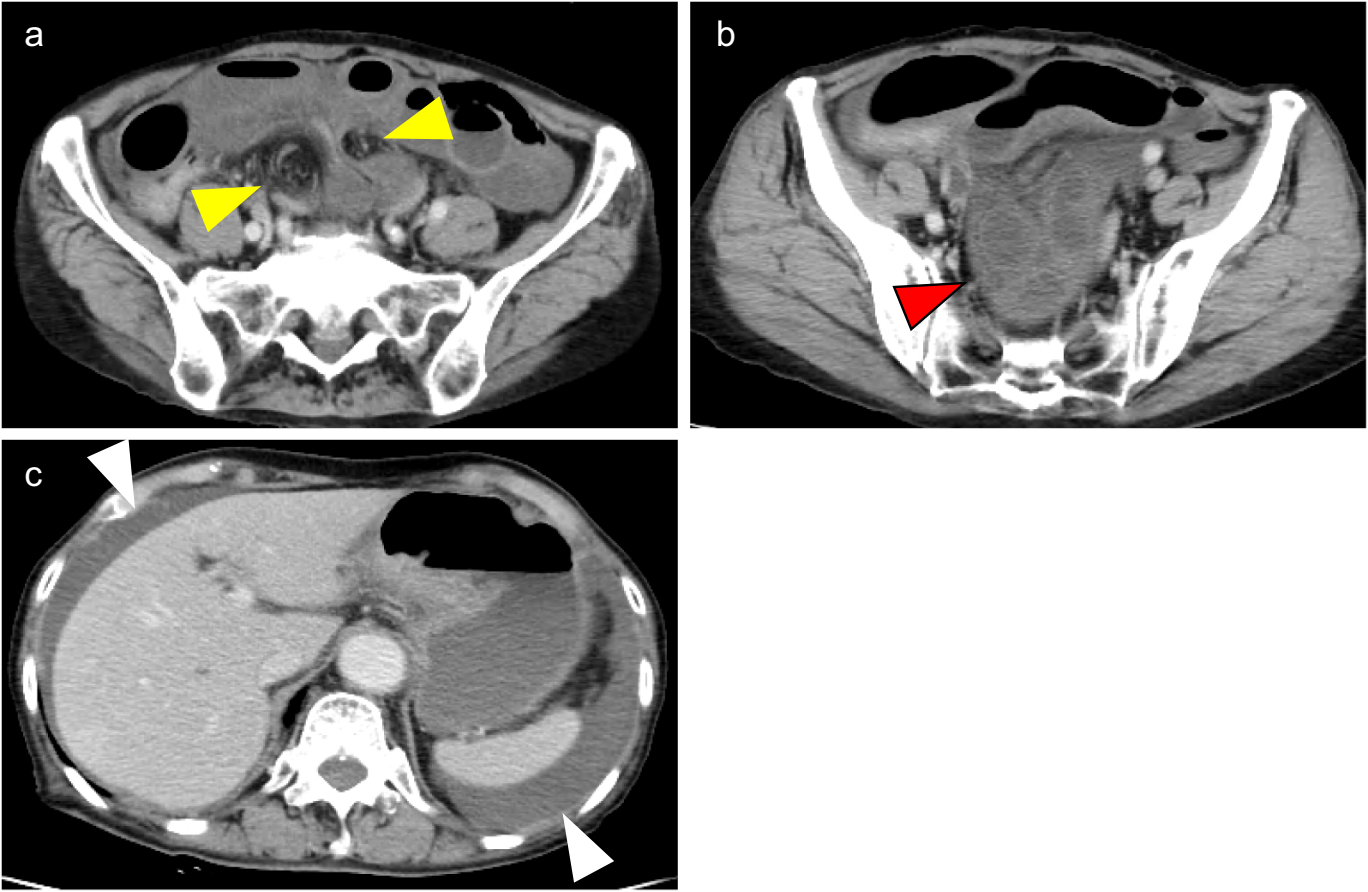
Contrast-enhanced CT imaging. **a** Hernia portal where the small bowel enters forming a closed loop (yellow arrow) **b** Dilated small bowel with poor contrast effect (red arrow) **c** Ascites (white arrow)

The patient was an 89-year-old woman who visited a neighborhood hospital 4 h after developing abdominal pain and vomiting of sudden onset. She was referred to our hospital 2 h later with the diagnosis of intestinal obstruction. She had a history of undergoing cesarean sections. On arrival at our hospital, her vital signs were stable; examination revealed that she was 145 cm tall and weighed 36.9 kg (calculated body mass index [BMI], 17.5). She had severe tenderness in the lower abdomen, but no signs of peritoneal irritation. Blood tests showed an elevated white blood cell count, although the serum C-reactive protein (CRP) was normal. Blood gas analysis showed mild acidosis, with a pH of 7.383, and a base excess (BE) of − 3.7. Contrast-enhanced computed tomography (CT) revealed a small bowel forming a closed loop, with poor contrast effect, and dilatation of the oral side of the small bowel (Fig. 1a, b). Ascites was also identified (Fig. 1c). The patient was diagnosed as having strangulated bowel obstruction, and emergency surgery was performed.

**Fig. 2:**
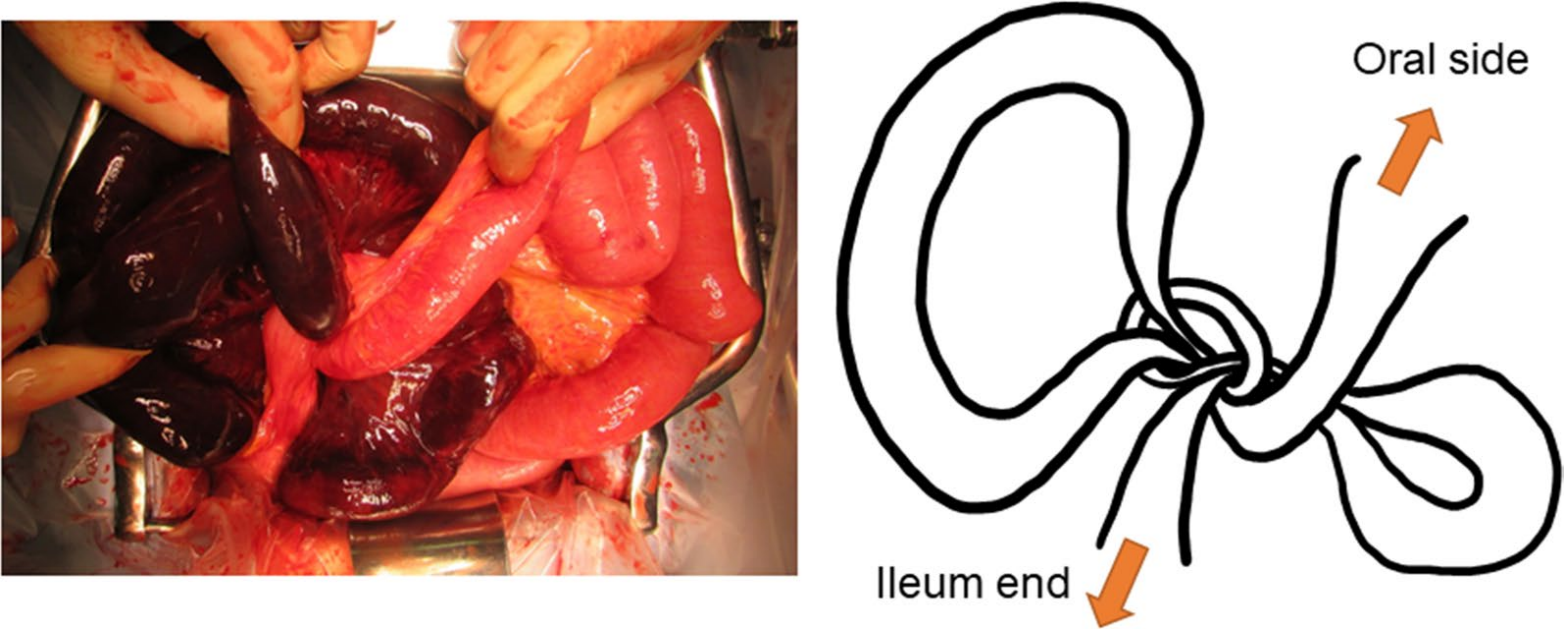
Intraoperative findings. Two segments of the ileum were tied together to form a knot, and both segments were necrotic

At laparotomy, bloody ascites was observed. Two segments of the ileum were tied together forming a knot, and both segments were necrotic due to impaired blood flow (Fig. 2). There was a band formation between a nearby segment of the small bowel and the abdominal wall, probably attributable in part to the previous cesarean sections, but there was no evidence of intestinal obstruction. Although it was necessary to release the strangulated small bowel, we did not immediately release the knot, but first proceeded with ligation of the mesenteric vessels draining the strangulated small bowel, to prevent dissemination of toxic substances from the necrotic bowel to the systemic circulation. Ligation of the mesenteric vessels was followed by resection of a 100-cm segment of the knotted necrotic ileum, 10 cm from the ileal end. Hand-sewn anastomosis was performed with the Albert-Lembert suture. The volume of blood loss was 282 ml, and the operation time was 1 h 41 min.

**Fig. 3:**
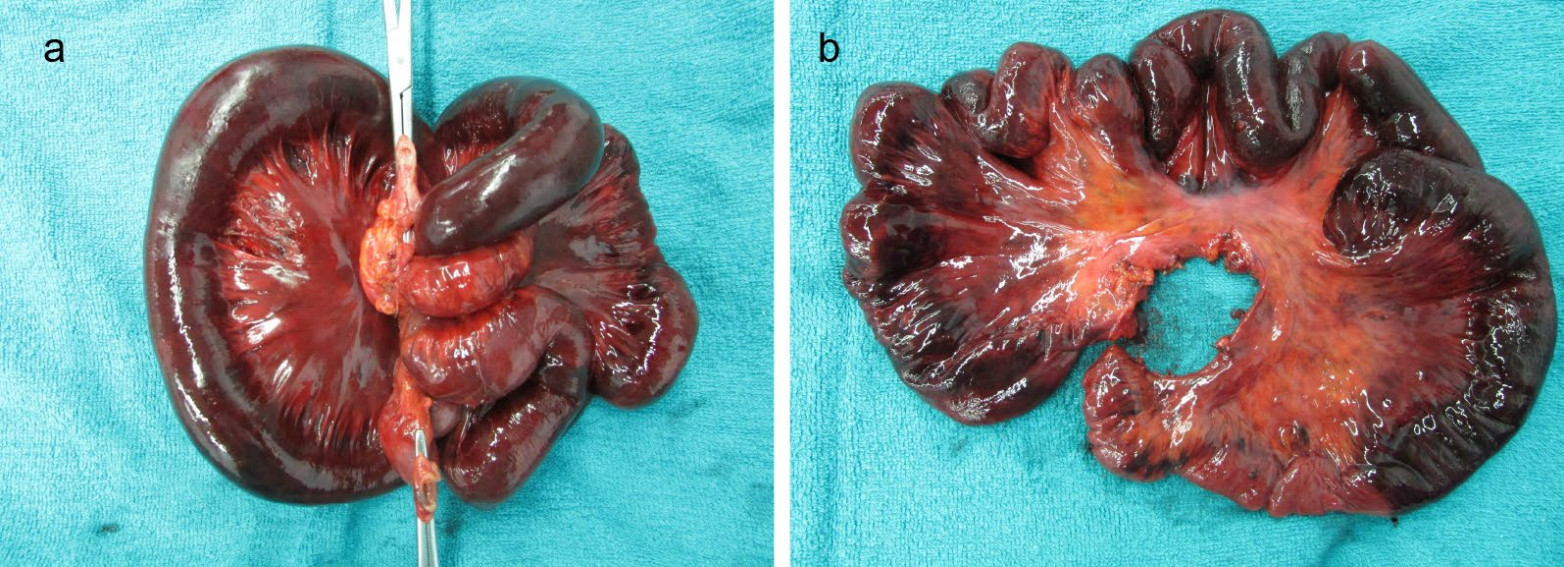
Findings of resected specimen. **a** Before releasing the knot. **b** After releasing the knot

The resected specimen showed the two intestinal segments wrapped together forming a knot, as indicated by the intraoperative diagnosis, and the strangulation was released by untying the knot (Fig. 3). There were no abnormalities on the mucosal surface other than signs of necrosis. Histopathological examination of the resected ileum showed that the resected small bowel was remarkably devoid of crypt epithelium, and there was severe congestion and hemorrhage extending from the intrinsic mucosal layer to the submucosa, which was considered to represent an ischemic change.

The postoperative course was uneventful, and the patient was discharged on the 13th postoperative day.

## Discussion

**Fig. 4:**
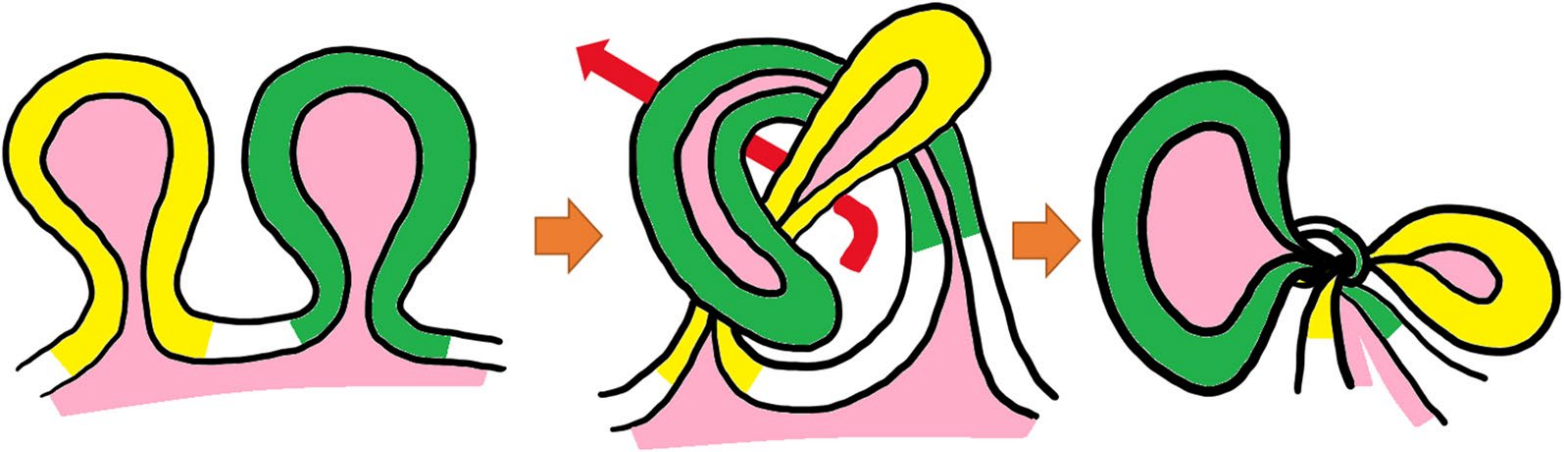
Shema of true knot. One intestinal segment (green) passes through the portal formed by the crossing of another intestinal segment (yellow)

Taylor et al. classified bowel knots into two types: true knots, in which one intestinal segment passes through a loop formed by another intestinal segment, and pseudo knots, in which the intestinal segments are wrapped around each other without actual formation of a knot [2]. The condition in the present case was classified as a true ileo-ileal knot, which is a rare cause of small bowel obstruction. The pathogenesis of strangulated bowel obstruction caused by pseudo knot formation is quite different from that associated with true knot formation, because adhesions are involved in the former. In this study, we focused on true knot formation, which is the narrow definition of intestinal knot formation (Fig. 4).

Intestinal obstruction caused by ileo-ileal knot formation is rare, and only 12 cases have been reported since 1990, including in the Japanese literature [1, 3–10] (Table 1). Considering the pathogenesis, the disease might be expected to be more common in older women with less visceral fat, but in the reported cases, it was also found to be quite common in younger people and in men. In almost all cases, the patients presented with abdominal pain and visited the hospital a relatively short time from the onset of the symptom. Surgery was also performed relatively early after the symptom onset, but most of the patients required small bowel resection due to intestinal necrosis. This may reflect the severe blood flow restriction caused by the knot formation. In the present case, we were able to perform surgery as early as 7 h after the symptom onset, but the small bowel was already necrotic.

**Table 1:** Details of 13 cases of ileo-ileal knot including our case

Author	Year	Age	Sex	Chief compliant	Surgical history	Preoperative diagnosis	Duration until surgery	Knot point	Operation	Untying before resection	Outcome
Kusaka [3]	1991	17	F	Abdominal pain	None	Appendicitis	18 h	30 cm from ileum end	Resection	Yes	Discharged on 29POD
Kondo [4]	1993	61	M	Abdominal pain	Gastrectomy	Strangulated bowel obstruction	3 h	Ileum	Resection	Yes	Discharged on survival
Kondo	1993	84	M	Abdominal pain, vomiting	None	Strangulated bowel obstruction	18 h	Ileum	Resection	N.A	Discharged on survival
Kondo	1993	76	F	N.A	Hysterectomy	Strangulated bowel obstruction	45 h	Ileum	Release obstruction	–	Discharged on survival
Kondo	1993	91	F	N.A	Common bile duct lithotripsy	Strangulated bowel obstruction	15 h	Ileum	Resection	N.A	Discharged on survival
Uday [5]	2012	68	M	Abdominal pain, vomiting	None	Strangulated bowel obstruction	48 h	15 cm from ileum end	Resection	Yes	Discharged on 8POD
Andromanakos [6]	2014	26	M	Abdominal pain	None	Strangulated bowel obstruction	6 h	Ileum	Resection	Yes	Discharged on 15POD
Abebe [7]	2015	55	F	Abdominal pain	None	Strangulated bowel obstruction	48 h	8 cm from ileum end	Resection	No	Discharged on 14POD
Uemura [8]	2016	90	M	Abdominal pain	Appendectomy	Strangulated bowel obstruction	< 24 h	5 cm from ileum end	Resection	Yes	Discharged on 19POD
Taniguchi [1]	2017	80	F	Abdominal pain	Colectomy, adnexectomy	Strangulated bowel obstruction	6 h	10 cm from ileum end	Resection	Yes	Discharged on 12POD
Matsuoka [9]	2017	27	M	Abdominal pain, vomiting	None	Strangulated bowel obstruction	8 h	Ileum	Resection	No	Discharged on 14POD
Rajesh [10]	2018	23	M	Abdominal pain, vomiting	Hirshsprung	Strangulated bowel obstruction	48 h	20 cm from ileum end	Release obstruction	–	Discharged on 8POD
Present case	2020	89	F	Abdominal pain, vomiting	Caesarean section	Strangulated bowel obstruction	7 h	10 cm from ileum end	Resection	No	Discharged on 13POD

*M* male, *F* female, *N.A.* not available

Although the intestinal tract entering another intestinal loop should be free of adhesions, about a half of the previously reported cases had a previous history of laparotomy. Kondo et al. suggested that adhesions may play a role in knot formation [4]. In our case, the oral side of the intestinal segment forming the knot was fixed by adhesions, and the anal side was fixed at the ileal end. Given the high incidence of knot formation near the ileal end, partial fixation of the bowel due to adhesions may be involved in the pathogenesis of knot formation. There are reports that intestinal knot formation is more common in countries with the custom of fasting and after gastrectomy, in which it may be triggered by the large amount of food flowing into the small intestine within a short period of time [8, 11]. We hypothesized that anchorage by moderate adhesions and exaggerated intestinal peristalsis could lead to accidental knot formation.

Preoperative diagnosis of knot formation is extremely difficult, and in most previously reported cases, the condition was only diagnosed as strangulated bowel obstruction prior to the operation. CT is often not performed in patients of advanced age or cases in developing countries. In our case, preoperative CT revealed the small bowel in a closed-loop formation, and the diagnosis of strangulated bowel obstruction was made; a retrospective review, however, confirmed two, not one, closed-loop formations. As Matsuoka et al. have pointed out previously [9], the “double closed-loop” formation could be considered as a unique finding in this disease.

True ileo-ileal knot formation cannot be prevented; therefore, immediate operation is required. In terms of the most appropriate surgical procedure, there is no consensus as yet as to whether the ileo-ileal knot should be released or not before resecting the bowel. Some suggest that the knot should be released first to determine the extent of salvageable small bowel, so as to avoid excessive bowel resection [8]. In fact, in 2 reported cases, the surgery was completed simply by only releasing the knot formation. Others, however, suggest that the knot should not be released so as to prevent contamination of the operative field and entry of necrotic material into the systemic circulation [7]. In our case, the necrosis and its extent were clear, and it was possible to resect the mesentery without releasing the knot. Since ileo-ileal knot is a type of strangulated bowel obstruction that requires attention to reperfusion dysfunction, we believe that it is better to perform mesenteric ligation prior to releasing strangulation.

There were no deaths among the cases included in this review. It is considered that appropriate surgery at the right time is associated with a good prognosis.

Because of small number of reported cases, we have very limited knowledge of true ileo-ileal knot. Furthermore, almost cases were reported from Japanese institute. True pathogenesis and appropriate surgical procedure for this disease should be assessed in the near future. Considering the background of lack of understanding of the disease, our case report may be helpful to understand the therapeutic strategy of true ileo-ileal knot.

## Conclusion

We encountered a case of strangulated small bowel obstruction caused by true ileo-ileal knot formation. Even though we were able to operate on the patient relatively early after the symptom onset, the involved bowel segment was found to be necrotic due to the severe blood flow restriction. Resection of the necrotic small intestine without releasing the knot might be considered as an option of surgical procedure.

## Data Availability

All data generated during this study are included in this published article.

## Abbreviations

BMI: Body mass index
CRP: C reactive protein
BE: Base excess
CT: Computed tomography
